# Aerobic Exercise Reduces Asthma Phenotype by Modulation of the Leukotriene Pathway

**DOI:** 10.3389/fimmu.2016.00237

**Published:** 2016-06-14

**Authors:** Ricardo Wesley Alberca-Custódio, Flávia Regina Greiffo, BreAnne MacKenzie, Manoel Carneiro Oliveira-Junior, Adilson Santos Andrade-Sousa, Gustavo Silveira Graudenz, Angela Batista Gomes Santos, Nilsa Regina Damaceno-Rodrigues, Hugo Caire Castro-Faria-Neto, Fernanda Magalhaes Arantes-Costa, Milton De Arruda Martins, Asghar Abbasi, Chin Jia Lin, Marco Idzko, Ana Paula Ligeiro Oliveira, Hinnak Northoff, Rodolfo Paula Vieira

**Affiliations:** ^1^Laboratory of Pulmonary and Exercise Immunology (LABPEI) and Brazilian Institute of Teaching and Research in Pulmonary and Exercise Immunology (IBEPIPE), Nove de Julho University (UNINOVE), São Paulo, Brazil; ^2^Laboratory of Cellular Biology (LIM 59), School of Medicine, University of São Paulo (USP), São Paulo, Brazil; ^3^Laboratory of Immunopharmacology, Institute Oswaldo Cruz (IOF), Oswaldo Cruz Foundation, Rio de Janeiro, Brazil; ^4^Laboratory of Experimental Therapeutics (LIM 20), School of Medicine, University of São Paulo (USP), São Paulo, Brazil; ^5^Institute for Memory Impairments and Neurological Disorders (MIND Institute), University of California Irvine, Irvine, CA, USA; ^6^Department of Pathology (LIM 05), School of Medicine, University of São Paulo (USP), São Paulo, Brazil; ^7^COPD and Asthma Research Group, Department of Pneumology, University Hospital Freiburg, Freiburg, Germany; ^8^Institute of Clinical and Experimental Transfusion Medicine (IKET), University of Tübingen, Tübingen, Germany

**Keywords:** airway inflammation, asthma, cytokines, exercise immunology, leukotrienes

## Abstract

**Introduction:**

Leukotrienes (LTs) play a central role in asthma. Low- to moderate-intensity aerobic exercise (AE) reduces asthmatic inflammation in clinical studies and in experimental models. This study investigated whether AE attenuates LT pathway activation in an ovalbumin (OVA) model of asthma.

**Methods:**

Sixty-four male, BALB/c mice were distributed into Control, Exercise (Exe), OVA, and OVA + Exe groups. Treadmill training was performed at moderate intensity, 5×/week, 1 h/session for 4 weeks. Quantification of bronchoalveolar lavage (BAL) cellularity, leukocytes, airway remodeling, interleukin (IL)-5, IL-13, cysteinyl leukotriene (CysLT), and leukotriene B4 (LTB4) in BAL was performed. In addition, quantitative analyses on peribronchial leukocytes and airway epithelium for LT pathway agents: 5-lypoxygenase (5-LO), LTA4 hydrolase (LTA4H), CysLT_1_ receptor, CysLT_2_ receptor, LTC4 synthase, and LTB4 receptor 2 (BLT2) were performed. Airway hyperresponsiveness (AHR) to methacholine (MCh) was assessed *via* whole body plethysmography.

**Results:**

AE decreased eosinophils (*p* < 0.001), neutrophils (*p* > 0.001), lymphocytes (*p* < 0.001), and macrophages (*p* < 0.01) in BAL, as well as eosinophils (*p* < 0.01), lymphocytes (*p* < 0.001), and macrophages (*p* > 0.001) in airway walls. Collagen (*p* < 0.01), elastic fibers (*p* < 0.01), mucus production (*p* < 0.01), and smooth muscle thickness (*p* < 0.01), as well as IL-5 (*p* < 0.01), IL-13 (*p* < 0.01), CysLT (*p* < 0.01), and LTB4 (*p* < 0.01) in BAL were reduced. 5-LO (*p* < 0.05), LTA4H (*p* < 0.05), CysLT_1_ receptor (*p* < 0.001), CysLT_2_ receptor (*p* < 0.001), LTC4 synthase (*p* < 0.001), and BLT2 (*p* < 0.01) expression by peribronchial leukocytes and airway epithelium were reduced. Lastly, AHR to MCh 25 mg/mL (*p* < 0.05) and 50 mg/mL (*p* < 0.01) was reduced.

**Conclusion:**

Moderate-intensity AE attenuated asthma phenotype and LT production in both pulmonary leukocytes and airway epithelium of OVA-treated mice.

## Introduction

Asthma is a chronic airway inflammatory disease affecting more than 300 million people around the world (1). Clinical manifestations of the disease include wheezing, breathlessness, chest tightness, cough, and variable airflow limitation (1). These manifestations are attributed to unresolved chronic airway inflammatory processes, leading to airway remodeling and hyperresponsiveness (AHR) (1, 2).

Leukotrienes (LTs) are bioactive eicosanoids derived from essential fatty acids and synthesized *via* the oxidation of arachidonic acid by leukocytes and airway epithelium (3–6). LTs are potent pro-inflammatory molecules, contributing to several aspects of asthma pathophysiology, including bronchoconstriction, edema formation, mucus hypersecretion, as well as inflammatory cell proliferation, activation, and survival (3–5, 7).

Leukotrienes are also centrally involved in exercise-induced bronchoconstriction (EIB) (8, 9). EIB is defined as a transient event marked by increased airway resistance, characterized by a decrease of 10% or more in forced expiratory volume in the first second (FEV_1_), a phenomenon triggered by high-intensity exercise (8–11). In fact, 70–90% of asthmatics, 40% of individuals with allergic rhinitis, and 5–10% of non-pulmonary and non-allergic patients present EIB (8–11). In addition, increased levels of LTs in urine and in breath condensate are commonly found after EIB in asthmatics (8–11). Cysteinyl leukotrienes (CysLTs), LTC_4_, LTD_4_, and LTE_4_ are the most important mediators involved in EIB (10, 11). However, a growing number of studies performed in animal models of asthma (12–17) as well as in the clinic (18–21) have demonstrated that in contrast to high-intensity aerobic exercise (AE), low- to moderate-intensity AE, significantly reduces flare-ups and leads to increased quality of life.

Though the mechanism by which moderate AE reduces airway inflammation is not completely understood, previous studies have demonstrated that AE reduces nuclear factor kappa light-chain enhancer of activated B cells (NF-κB) activation and increases release of anti-inflammatory cytokine interleukin (IL)-10 (12–17). In mice, chronic ovalbumin (OVA) exposure reproduces some hallmarks of human allergic asthma, including allergen-dependent sensitization, a Th2-dependent allergic inflammation characterized by eosinophilic influx into the airway mucosa, and AHR (22). Both leukocytes and stimulated airway epithelium produce LTs, which contribute to NF-κB activation and drive the inflammatory process in OVA-stimulated mice (23). Therefore, the aim of this study was to test whether moderate AE attenuates LT pathway activation in the OVA model, as LT signaling is an important contributor to inflammation in both allergic asthma and EIB.

## Materials and Methods

All experimental procedures were approved by the ethical committee from Nove de Julho University (UNINOVE), protocol AN0021/2013, and were carried out in accordance to Declaration of Helsinki 2013.

### Animals and Experimental Groups

Sixty-four male Balb/c mice (18–22 g) were distributed into Control (Control; non-manipulated), Exercise (Exe; only exercised), OVA (OVA; sensitized and challenged with OVA), and OVA + Exercise (OVA + Exe; sensitized and challenged with OVA and exercised) groups. The study was performed twice such that two groups of eight animals/group were included.

### Experimental Model of Asthma

**Figure 1:**
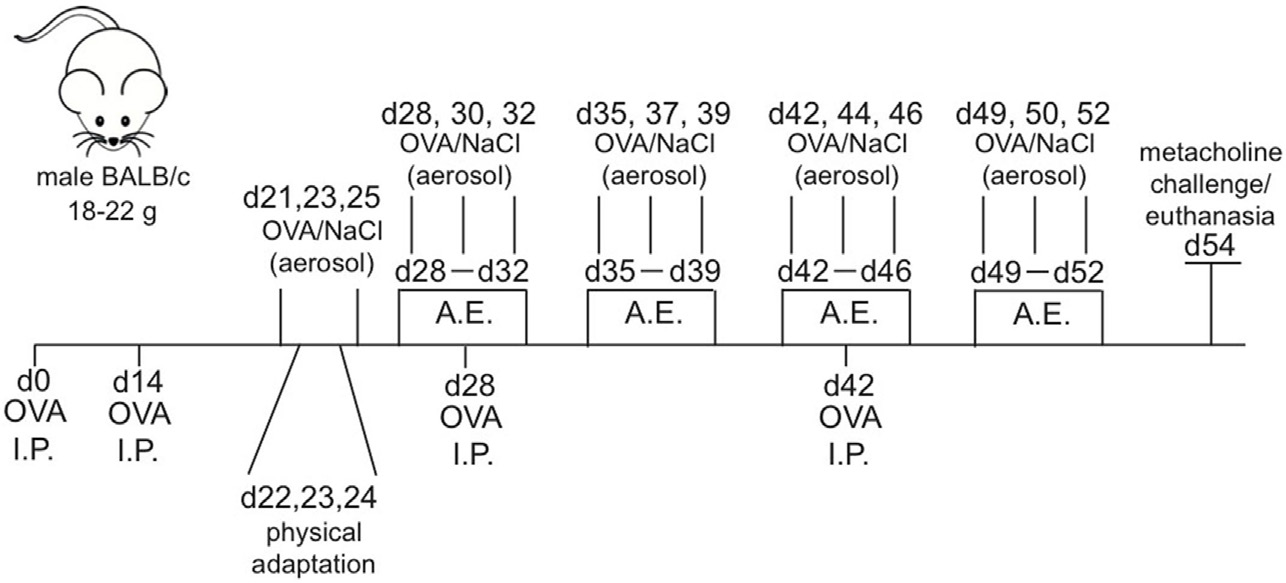
**Allergic asthma and aerobic exercise experimental model**. Male Balb/c mice (*n* = 8/group) received OVA i.p. injections (20 μg) on day 0, 14, 28, and 42. The 30-min aerosol treatments for OVA (1%) were given 3 days a week starting from day 21. Aerobic exercise training was performed 30 min a day, 5 days a week starting from day 28 and ending on day 52. Euthanasia and/or metacholine challenge (25 and 50 mg/mL) was performed on day 54.

Animals belonging to OVA and OVA + Exe groups were sensitized on days 0, 14, 28, and 42 using OVA (20 μg/mouse) absorbed in aluminum hydroxide diluted in sterile 0.9% NaCl solution. Aerosol challenges (1% OVA solution) were performed for 30 min/session, 3×/week, beginning on day 21 and continued until day 53. Control groups were subjected to the same protocol using only 0.9% NaCl solution (12). See Figure 1 for a detailed schematic.

### Treadmill Physical Training

The low intensity treadmill training (0.7 km/h, 25% inclination) began on day 26 of experimental protocol and continued until day 54 and was performed 5×/week, 60 min/session (12). See Figure 1 for a detailed schematic.

### Bronchoalveolar Lavage

Mice were anesthetized (10 mg/kg ketamine and 100 mg/kg xylazin intra-peritoneal), and bronchoalveolar lavage (BAL) was harvested, prepared, and analyzed, as described previously (12).

### Cysteinyl Leukotrienes, Leukotriene B4, and Th2 Cytokines Measurement in BAL

The levels of IL-5 and IL-13 in BAL fluid were measured using ELISA kit from Biolegend (CA, USA), whereas CysLT and leukotriene B4 (LTB4) in BAL were measured using Parameter Assay Kit from R&D Systems (IL, USA), following manufacturer’s instructions.

### OVA-Specific Antibody Measurements in Serum

IgG1 (Th2), IgG2 (Th1), and IgE antibody production were measured using ELISA technique, as described previously (24).

### Quantitative Analysis of Airway Inflammation and Remodeling

The lungs were removed *in block*, fixed in 4% formalin solution, and submitted to classical histological analysis, followed by staining of 4 μm tissue slices with hematoxylin and eosin for density analyses of eosinophils, lymphocytes, and macrophages in the airway wall. Airway smooth muscle thickness was visualized by picrosirius staining, and the density of collagen fibers in airway walls was visualized by resorcin–fuchsin with oxidation (for determination of the density of elastic fibers in airway walls). Finally, periodic acid Schiff with alcian blue (PAS/AB) staining was performed for the visualization of acidic and neutral mucus content in the airway epithelium. Five random airways per animal were photographed using an optical microscope Olympus BX40 (Olympus, PA, USA) and digital camera QColor 5 (Olympus, PA, USA). The analyses were performed using standard image analysis protocol using the software CellSens (Olympus, PA, USA) as follows: to calculate the density of eosinophils, lymphocytes, and macrophages, peribronchial areas were delimited (defined as the areas between airway epithelial basal membrane and airway adventitia), and cells were counted according to their morphological criteria. The results were expressed as number of cells per square millimeter.

Collagen fiber density was measured between the airway epithelial basal membrane and airway adventitia. Collagen fibers were calculated as % of total collagen using a CellSens software color threshold.

Epithelial mucus content was measured between the airway epithelial basal membrane and the inner epithelium layer of airway. The neutral and acidic mucus content was determined using CellSens software color threshold.

### Immunohistochemistry

Immunohistochemistry was performed on 4 μm tissue slices in slides previously prepared with silane. The slices were incubated with primary antibodies anti-5-lypoxygenase (5-LO), anti-LTA4 hydrolase (LTA4H), anti-CysLT_1_ receptor, anti-CysLT_2_ receptor, anti-LTC4 synthase, anti-LTB4 receptor 2 (BLT2) (Santa Cruz Biotechnology, Santa Cruz, CA, USA), and with proper secondary antibodies conjugated with biotin–streptavidin–peroxidase. The number of positive peribronchial leukocytes, as well as the percentage of epithelial area expressing each LT pathway marker was performed using a combined image analysis with point-counting technique (12, 13). Counting was performed in five airways for each animal of all experimental groups at 400× magnification. Stained peribronchial positive leukocytes were expressed as the number of positive cells per square millimeter (12, 13), whereas airway epithelium protein expression was represented as a percentage of positive area of the epithelium.

### Airway Hyperresponsiveness

The responsiveness to methacholine (MCh) was assessed in conscious mice using the whole body plethysmograph (Buxco Europe, Winchester, UK), as described previously (25, 26). Enhanced pause (Penh) has a theoretical relationship with airway obstruction and its response to growing doses of MCh (6.25, 12.5, 25, and 50 mg/mL) was used as an index of AHR (25, 26).

### Statistical Analysis

Statistical analysis was performed using Graph Pad Prism 5.0. Normality analysis revealed parametric data that were expressed as mean ± SD. Comparisons between groups were carried out by two-way analysis of variance (ANOVA), followed by Student–Newman–Keuls *post hoc* test. Values were considered significant at *p* < 0.05.

## Results

### Aerobic Exercise Attenuated Airway Inflammation in OVA-Treated Mice

**Figure 2:**
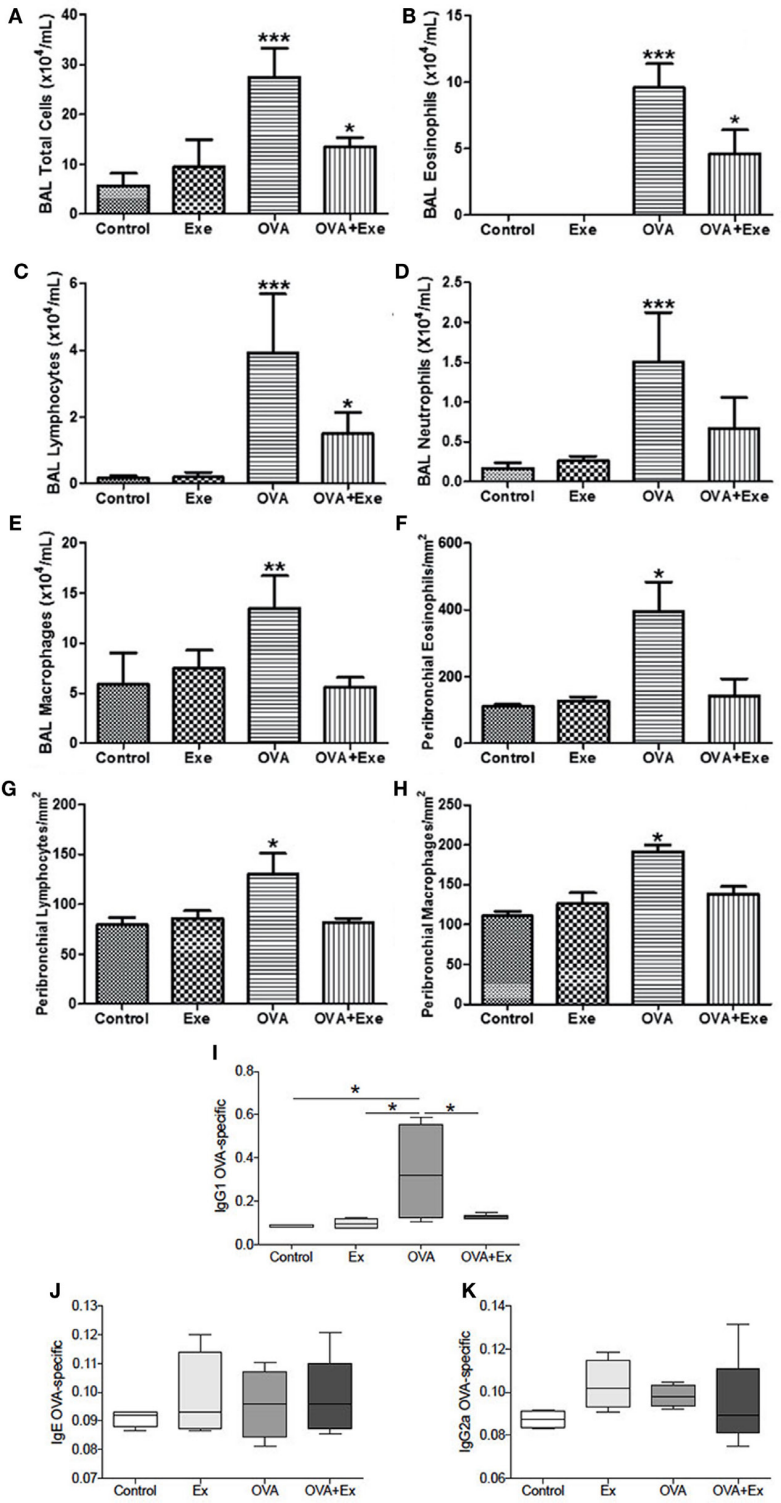
**Aerobic exercise decreased the number of leukocytes in BAL and in airway wall**. Total cells **(A)**, eosinophils **(B)**, lymphocytes **(C)**, neutrophils **(D)**, and macrophages **(E)** from bronchoalveolar lavage, and also the number of eosinophils **(F)**, lymphocytes **(G)**, and macrophages **(H)** in the airways wall. IgG1 OVA-specific antibody was increased in OVA and decreased in OVA + Exe **(I)**. No regulation was observed for OVA-specific IgE **(J)** or IgG2a **(K)**. The results are expressed as mean ± SE. For **(A–D)**, ***p < 0.001 compared with all groups. For **(E)**, **p < 0.01 compared with all groups. For **(F–H)**, *p < 0.05 compared with all groups. For **(A–C)**, *p < 0.05 compared with control group. For **(I)**, *p < 0.05.

The allergic asthma and AE protocol were performed, as described previously (13); a schematic of the protocol is provided (Figure 1). In order to confirm the presence of robust inflammation due to OVA-induced chronic allergic airway inflammation, the total and differential number of eosinophils, lymphocytes, and macrophages were measured in BAL, and also in the airway wall of Control, Exercise, OVA, and OVA + Exe groups. OVA exposure resulted in a significant influx of leukocytes in BAL; total cells (Figure 2A), eosinophils (Figure 2B), lymphocytes (Figure 2C), neutrophils (Figure 2D), and macrophages (Figure 2E), and also in airway wall: eosinophils (Figure 2F), lymphocytes (Figure 2G), and macrophages (Figure 2H). On the other hand, AE significantly reduced the number total cells (Figure 2A), eosinophils (Figure 2B), lymphocytes (Figure 2C), neutrophils (Figure 2D), and macrophages (Figure 2E) in BAL, as well as the number of eosinophils (Figure 2F), lymphocytes (Figure 2G), and macrophages (Figure 2H) in the airway wall. In addition, OVA-specific antibody production in the serum was measured by ELISA. Th2-specific IgG1 antibody was significantly increased in OVA mice and attenuated in OVA + Exe animals (Figure 2I). However, IgG2 (Th1-specific antibody) and IgE were not regulated in this model (Figures 2J,K).

### Aerobic Exercise Reduced Airway Remodeling in OVA-Treated Mice

**Figure 3:**
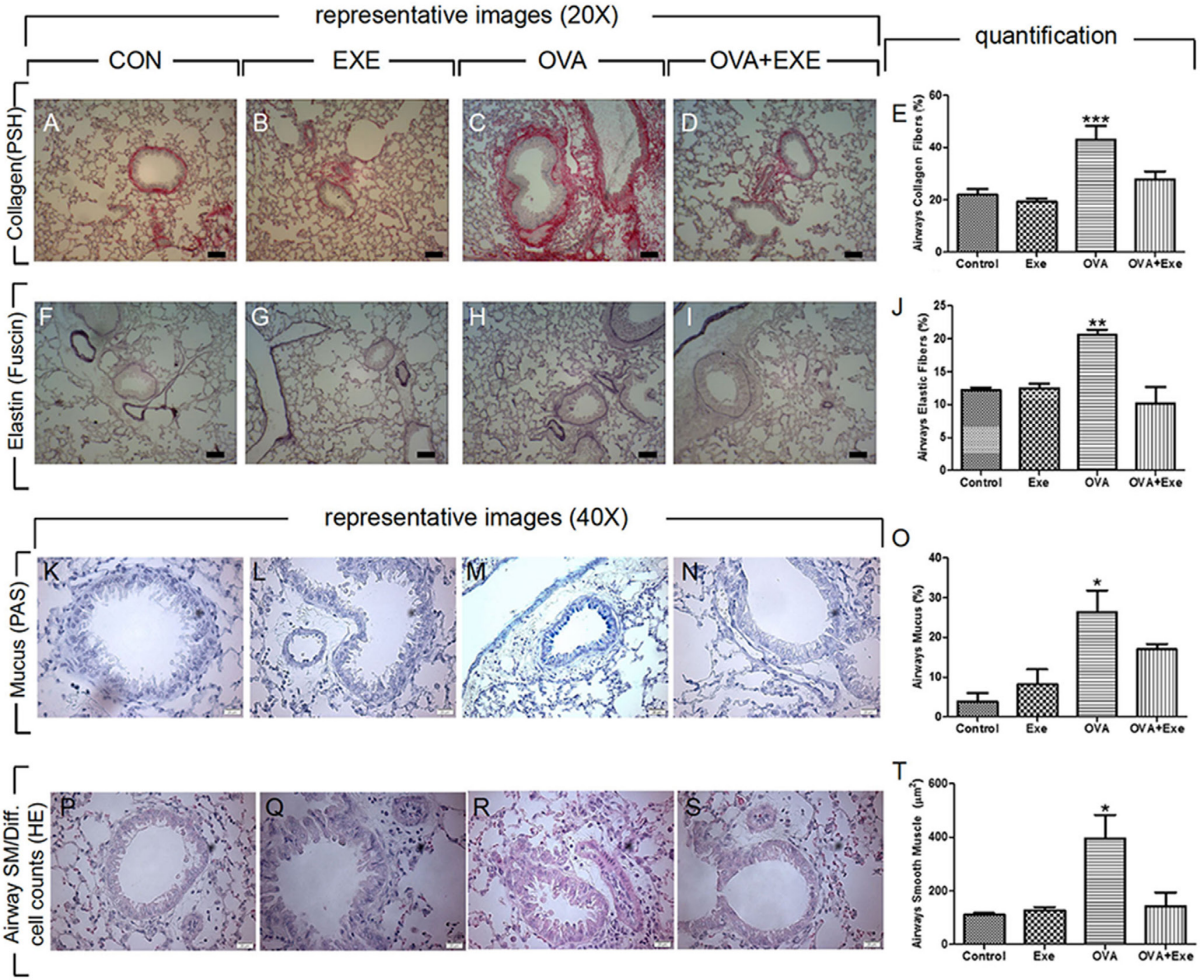
**Aerobic exercise attenuated OVA-induced remodeling**. Collagen fiber deposition visualized by picrosirius red **(A–D)** percentage of airway collagen fibers were calculated in *n* = 4/group **(E)**. Airway elastic fiber deposition visualized by resorcin–fuchsin staining **(F–I)** and percentage of elastic fibers deposition in the airway walls were calculated in *n* = 4/group **(J)**. Airway mucus production were visualized by periodic acid Schiff with alcian blue staining **(K–N)** and percentage of airway mucus in bronchial epithelium was quantified **(O)**. Finally, airway smooth muscle thickness was visualized by H + E staining **(P–S)** and airway smooth muscle thickness (micrometers) was quantified **(T)**. The results are expressed as mean ± SE. For Figure 2A, ***p < 0.001 compared with all groups. For Figure 2B, **p < 0.01 compared with all groups. For Figures 2C,D, *p < 0.05 compared with all groups.

To confirm airway remodeling due to chronic OVA exposure, airway collagen and elastic fibers accumulation, as well as airway mucus production and the thickness of airway smooth muscle of control, exercise, OVA, and OVA + Exercise groups were analyzed. Quantification of immunostainings was performed on at least four animals per group and representative images are shown. As reported previously, OVA exposure resulted in increased accumulation of collagen (Figures 3A–E) and elastic fibers (Figures 3F–J) in the airway walls. Likewise, airways mucus production (Figures 3K–O) and airway smooth muscle thickness (Figures 3P–T) were also increased in OVA-exposed mice. Notably, moderate AE significantly reduced OVA-stimulated accumulation of collagen (Figures 3D,E) and elastic fibers (Figures 3I,J) in the airway walls, as well as airway mucus production (Figures 3N,O) and airway smooth muscle thickness (Figures 3S,T).

### CystLTs, LTB4, and Th2 Cytokines Levels Were Significantly Reduced in the BAL Fluid of Exercised Mice

**Figure 4:**
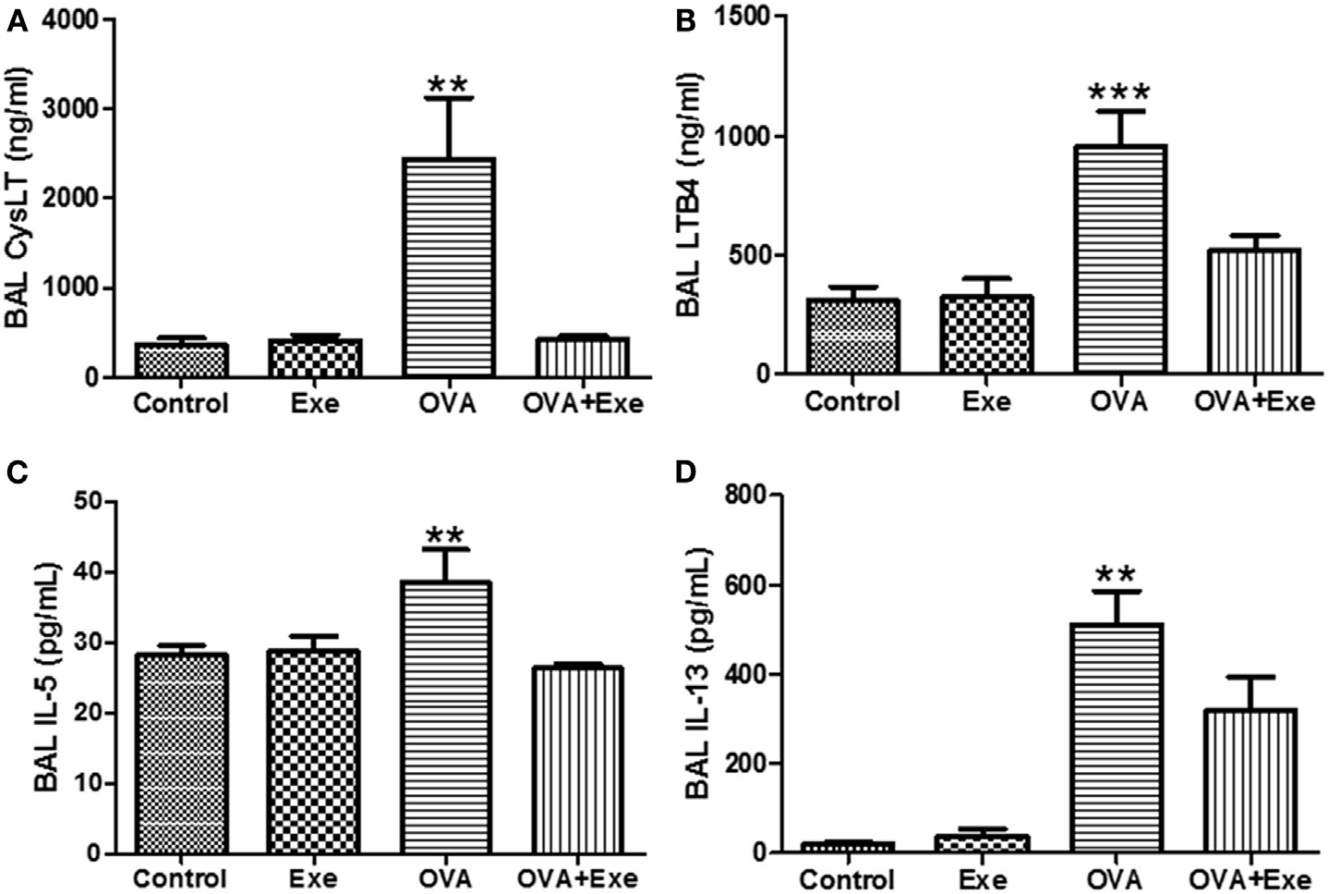
**CystLTs and pro-inflammatory cytokines were reduced in the BAL of exercised OVA mice**. ELISA measurements of total CystLTs **(A)**, LTB4 **(B)**, IL-5 **(C)**, and IL-13 **(D)**. The results are expressed as mean ± SE. For **(A,C,D)**, **p < 0.01 compared with all groups. For **(B)**, **p < 0.01 compared with all groups.

Given that LTs contribute to NF-κB activation, which stimulates the inflammatory process in OVA-stimulated mice (23), this study hypothesized that one of the mechanisms by which moderate-intensity AE reduces OVA-induced inflammation is by attenuating the LT pathway. Indeed, total CysLTs (Figure 4A) and leukotriene B4 (LTB4) (Figure 4B) as well as Th2 pro-inflammatory cytokines IL-5 (Figure 4C) and IL-13 (Figure 4D) were significantly increased in the BAL of OVA-exposed mice. On the other hand, total CystLTs (Figure 4A) and leukotriene B4 (LTB4) (Figure 4B) as well as pro-inflammatory cytokines IL-5 (Figure 4C) and IL-13 (Figure 4D) were significantly decreased in the BAL of OVA + Exe mice.

### Exercise Reduced the Expression of 5-LO, CysLT1R, CysLT2R, LTB4R2, LTA4H, and LTC4S by Airway Epithelial Cells and by Peribronchial Leukocytes

**Figure 5:**
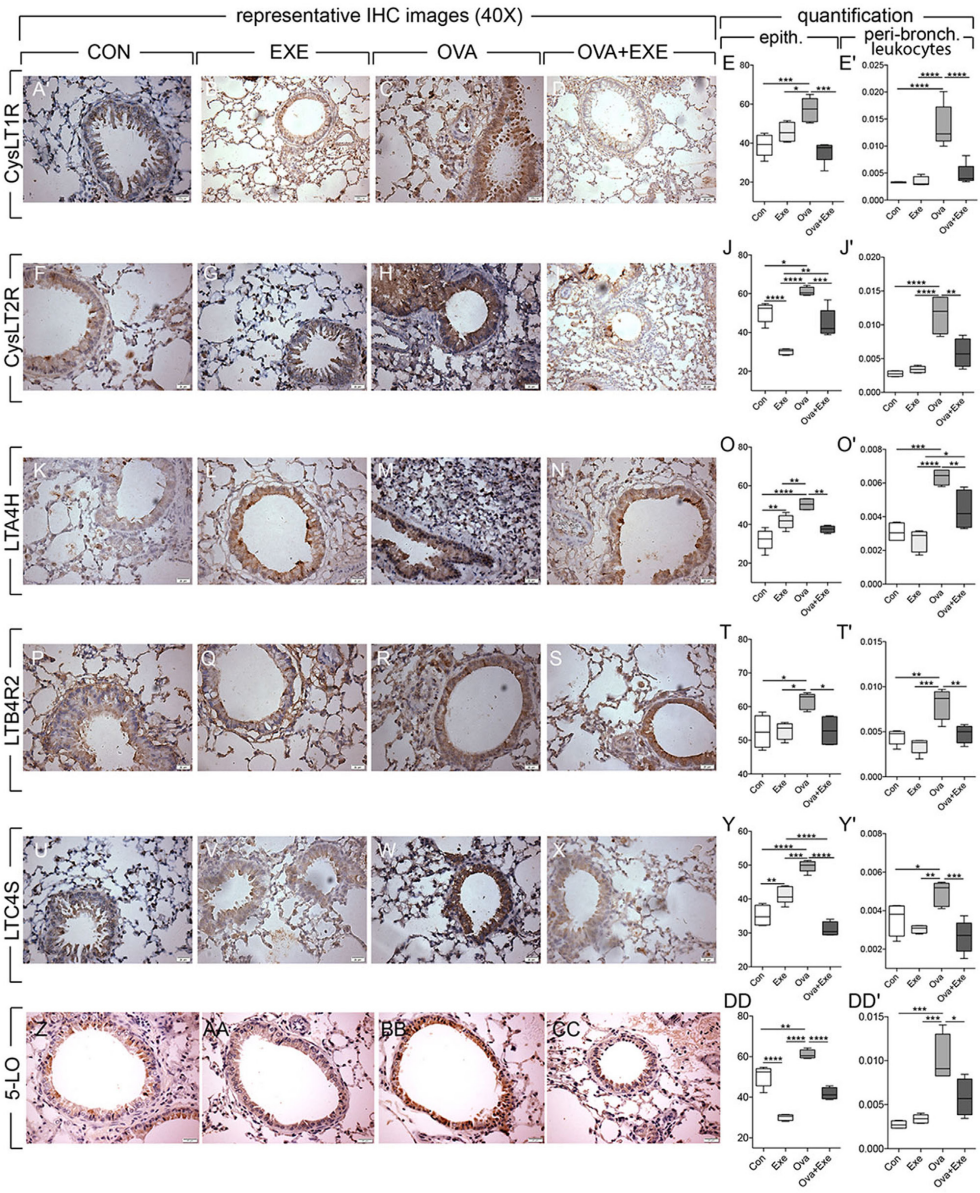
**Reduced epithelial expression of leukotriene pathway markers in exercised OVA mice**. Figure shows airway epithelium expression of CysLT1R **(A–D)**, CysLT2R **(F–I)**, LTA4H **(K–N)**, LTB4R2 **(P–S)**, LTC4S **(U–X)**, and 5-LO **(Z–CC)**. Quantification of epithelial staining was performed on at least five animals and five random areas per animal. Percentage of positive epithelial cells/area and positive peribronchial leukocytes/area were calculated for CysLT1R **(E,E’)**, CysLT2R **(J,J’)** LTA4H **(O,O’)**, LTB4R2 **(T,T’)**, LTC4S **(Y,Y’)**, and 5-LO **(DD,DD’)**. Results are expressed as mean ± SE and *p < 0.05, **p < 0.01, ***p < 0.001, and ****p < 0.0001. For 5-LO, OVA increased **p < 0.01 compared with all other groups.

In order to determine in which cell types AE reduces LT signaling in OVA-stimulated mice, immunolocalization LT pathway proteins were performed in airway epithelial cells and in peribronchial leukocytes. Quantitative analysis of immunohistochemistry was performed on at least four animals per group in both epithelial and peribronchial leukocytes for 5-LO, CysLT1R, CysLT2R, LTB4R2, LTA4H, and LTC4S. Representative images are shown. OVA exposure significantly increased the expression of 5-LO, CysLT1R, CysLT2R, LTB4R2, LTA4H, and LTC4S by airway epithelial cells and by peribronchial leukocytes (Figure 5). In contrast, OVA + Exe mice exhibited significantly reduced both epithelial and peribronchial leukocyte expression of LT pathway markers: CysLT1R (Figures 5A–E’), CysLT2R (Figures 5F–J’), LTA4H (Figures 5K–O’) LTB4R2 (Figures 5P–T’), LTC4S (Figures 5U–Y’), and 5-LO (Figures 5Z–DD’).

### Airway Hyperresponsiveness Was Attenuated by Aerobic Exercise

**Figure 6:**
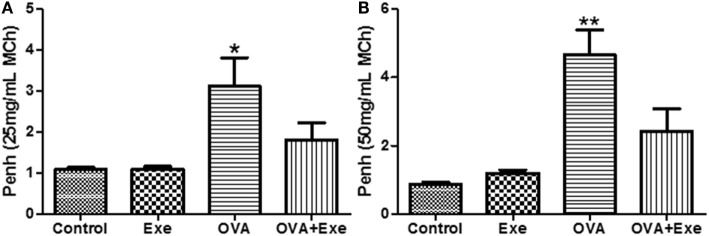
**Figure shows the airway hyperresponsiveness (AHR) for methacholine (MCh) 25 mg/mL (A) and for 50 mg/mL (B) from control, exercise, OVA, and OVA + Exercise groups**. The AHR was evaluated through whole body plethysmography (Buxco).

Airway hyperresponsiveness is a hallmark of asthma. In this study, OVA exposure resulted in a marked increase in AHR for MCh 25 mg/mL (Figure 6A) and 50 mg/mL (Figure 6B), whereas for PBS, 6.25 and 12.5 mg/mL no differences were found (data not shown). Importantly, AE significantly reduced AHR to MCh 25 mg/mL (Figure 6A) and 50 mg/mL (Figure 6B), demonstrating that the anti-inflammatory and the anti-fibrotic effects of AE are followed by reduction in AHR.

## Discussion

This study demonstrated for the first time that moderate-intensity AE decreased LT pathway activation in both airway epithelium and in peribronchial leukocytes. While previous animal studies have demonstrated that moderate AE reduces allergic asthma features, such as airway inflammation, exacerbated Th2 immune response, airway remodeling, and AHR, the results of this study suggest for the first time that these outcomes are at least partially linked to the ability of moderate AE to deactivate the LT pathway.

A growing number of both clinical and animal studies have demonstrated that the beneficial effects of AE on asthmatic phenotypes include anti-inflammatory and anti-fibrotic effects as well as the inhibition of AHR (3, 16, 18–20, 24, 28, 29, 31, 32), NF-κB inhibition (20, 29, 31, 32), Th2 immune response inhibition (16, 20, 28, 29, 31, 32), increased number of T-regulatory cells (15, 25), increased endogenous glucocorticoids production, and increased glucocorticoids receptor expression in the lungs (14, 18). In agreement with these studies, this study showed that in the context of OVA, moderate AE inhibited eosinophilic inflammation, as demonstrated through reduced number of eosinophils in BAL and in the airway walls, as well as the Th2 immune response, as IL-5 and IL-13 levels were reduced in BAL.

Aerobic exercise also exerts inhibitory effects on airway remodeling by inhibiting mucus synthesis, smooth muscle thickness, and elastic and collagen fiber deposition (16, 20, 24, 25, 28). This study clearly confirms such effects, since AE inhibited OVA-induced collagen and elastic fibers deposition in airway walls and also smooth muscle thickness and mucus synthesis. These structural changes in the airways are classically linked to the development and the severity of AHR.

Airway hyperresponsiveness is a key feature of asthma and can be induced in experimental models of asthma (13, 29). Similar to previous studies, this study demonstrated OVA-induced AHR due to MCh. Chronic, moderate-intensity AE efficiently inhibited OVA-induced AHR, further supporting the hypothesis that low to moderate AE may inhibit not only airway inflammation and remodeling but also AHR, an important functional aspect of asthmatic airways. This inhibitory effect of AE on AHR may be attributed to the extended or “chronic” exercise routine to which mice in this study were subjected to previous studies demonstrated that while single bouts of AE did not inhibit AHR (30), repeated bouts did (29). These experimental findings are confirmed by a recent study showing that chronic AE may inhibit AHR (14, 18, 26–29).

Airway hyperresponsiveness due to exercise, commonly referred to as EIB, is highly prevalent in asthmatics (70–90%) (1, 2, 4, 14). Several studies implicate increased levels of CysLTs as the most important mediator involved in EIB (10, 11). The LTs molecules, LTC_4_, LTD_4_, and LTE_4_ are collectively termed CysLTs and are involved not only in AHR but also in the severity of the inflammatory and remodeling process in asthma (31). CysLTs antagonists have been successfully targeted for asthma therapies (25, 32, 33) and as well as EIB (8, 9), especially for asthmatic non-responders to corticosteroid therapy (4, 31). Pharmacologically, LT activation can be inhibited *via* receptor antagonists (zafirlukast, pranlukast, and montelukast) or biosynthesis inhibition (Zileuton) (4, 31). Therefore, this literature suggests that the inhibition of LT receptors or their synthesis results in improved asthma control due to decreased airway inflammation and AHR (3, 4, 31).

This study demonstrated for the first time that AE inhibited not only the expression of LT receptors but also molecules important for LT biosynthesis. Furthermore, the study showed that the inhibitory effects of AE on the LT pathway occurred not only in peribronchial leukocytes but also in airway epithelial cells, resulting in a diminished accumulation of CysLTs and LTB4 in OVA + Exe lungs. These findings are particularly important, as both leukocytes and airway epithelial cells modulate several aspects of LT synthesis and action (4, 31). However, this study is limited in that it does not establish a direct, causal relationship between the observed beneficial effects of AE on OVA lungs due to exercise-induced attenuation of the LT pathway. Further studies using OVA-exercise models on mice with genetically amenable LT pathways may further elucidate the extent by which moderate AE contributes to reducing asthma-like symptoms by subduing LT pathway activation.

In summary, this study provides evidence that moderate-intensity AE inhibits airway inflammation, remodeling, and AHR in a model of OVA-induced asthma, by attenuating the expression of LT receptors and LT biosynthesis.

## Author Contributions

RC, FG, BM, and MO-J contributed to the preparation of the manuscript. MO-J also performed ELISA. AA-S performed IHC. GG and AS cared for the animals and performed the OVA injury. ND-R, HC-F-N, MM, AA, and CL contributed to the experimental design. MI, AO, HN, and RV contributed to the experimental design and interpretation of the results.

## Conflict of Interest Statement

The authors declare that the research was conducted in the absence of any commercial or financial relationships that could be construed as a potential conflict of interest.
